# Automated Characterization of Abdominal MRI Exams Using Deep Learning

**DOI:** 10.21203/rs.3.rs-5334453/v1

**Published:** 2024-12-09

**Authors:** Joonghyun Kim, Allison Chae, Jeffrey Duda, Ari Borthakur, Daniel Rader, James C. Gee, Charles E. Kahn, Penn Medicine BioBank, Walter R. Witschey, Hersh Sagreiya

**Affiliations:** University of Pennsylvania; University of Pennsylvania; University of Pennsylvania; University of Pennsylvania; University of Pennsylvania; University of Pennsylvania; University of Pennsylvania; University of Pennsylvania; University of Pennsylvania; University of Pennsylvania

## Abstract

Advances in magnetic resonance imaging (MRI) have revolutionized disease detection and treatment planning. However, as the volume and complexity of MRI data grow with increasing heterogeneity between institutions in imaging protocol, scanner technology, and data labeling, there is a need for a standardized methodology to efficiently identify, characterize, and label MRI sequences. Such a methodology is crucial for advancing research efforts that incorporate MRI data from diverse populations to develop robust machine learning models. This research utilizes convolutional neural networks (CNNs) to automatically classify sequence, orientation, and contrast, specifically tailored for abdominal MRI. Three distinct CNN models with similar backbone architectures were trained to classify single image slices into one of 12 sequences, 4 orientations, and 2 contrast classes. Results derived from this method demonstrate high levels of performance for the three specialized CNN models, with model accuracies for sequence, orientation, and contrast of 96.9%, 97.4%, and 97.3%, respectively.

## Introduction

Magnetic Resonance Imaging (MRI) is widely used in medicine, providing insights into anatomical structures, tissue characteristics, and pathological conditions. MRI is often used as a problem-solving modality, providing a more definitive diagnosis for equivocal findings seen on CT and ultrasound [1]. Moreover, it is used to aid in the diagnosis of a wide range of diseases such as intracranial tumors, traumatic brain injury (TBI), developmental anomalies, multiple sclerosis, stroke, and dementia [2]. As part of MRI imaging protocols, various sequence types, orientations, and patterns of contrast enhancement are used, which can vary widely by MRI scanner and institution [3]. Radiology reports provided to clinicians offer precise delineation of the most salient tissue characteristics and physiological processes.

Each MRI sequence highlights specific tissue characteristics, and combining data from multiple sequences allows radiologists to narrow the differential diagnosis. MRI pulse sequences are affected by repetition time (TR) and echo time (TE). TR refers to the interval between consecutive RF excitation pulses, while TE denotes the duration from the RF excitation to the echo detection [4]. TR is often used to control the T1 weighting of an image, while TE is used to determine the amount of T2 weighting [5]. T1-weighted sequences generally consist of short TR and TE, while T2-weighted sequences consist of long TR and long TE; structures that are hyperintense on T1-weighted sequences include fat, protein, certain phases of hemorrhage, melanin and gadolinium contrast, while fluid-containing/edematous structures are hyperintense on T2-weighted imaging [6]. Sequences with fat saturation signals are designed to selectively suppress the signal from fat, while preserving the signal from water and other tissues. MRCP sequences are highly T2-weighted and are hence specifically tailored for visualizing the biliary tree and pancreatic duct [7]. Diffusion-Weighted Imaging (DWI), in conjunction with ADC (Apparent Diffusion Coefficient) mapping, are crucial for assessing tissue microstructure, particularly in the setting of cancer, and detecting acute ischemic stroke [8]. T1-weighted gradient echo in-phase and out-of-phase sequences can aid in identifying fat, iron, and air [9]. Contrast-enhanced sequences can aid in medical diagnosis by highlighting differential perfusion characteristics that are representative of certain clinical entities, such as hepatic hemangioma versus focal nodular hyperplasia. Subtraction sequences enhance the detection of dynamic tissue changes by subtracting pre-contrast images from post-contrast images. Bright blood imaging sequences emphasize vascular structures. The care bolus sequence is used to aid in contrast agent timing. Finally, localizer sequences assist in precise patient positioning and scan planning.

Furthermore, MRI images can be acquired in various orientations–most commonly axial, sagittal, and coronal–depending on clinical requirements and the anatomical region of interest. The choice of orientation impacts the visualization of anatomical structures and facilitates accurate localization and characterization of pathological findings. As described above, the presence or absence of contrast enhancement plays a pivotal role in MRI interpretation, with contrast agents such as gadolinium enhancing tissue conspicuity and aiding in the detection and characterization of lesions, vascular abnormalities, and tumor viability [10].

Despite the diagnostic utility of MRI, inconsistencies in imaging protocols, interpretation practices, and reporting standards pose significant challenges to the standardization and reproducibility of MRI interpretation across healthcare institutions and radiology departments [11]. This can make the interpretation of outside MRI examinations a particular challenge. Variations in imaging parameters, scanner technology, and radiologist expertise can introduce discrepancies in image interpretation, potentially leading to diagnostic errors, delays in patient care, and inconsistencies in clinical decision-making.

In response to these challenges, automated methods for identifying MRI sequences have emerged as promising solutions to enhance efficiency and standardization in medical imaging interpretation. Prior methods focused primarily on brain MRI sequence classification [12, 13, 14, 15]. A study by Braeker et al. (2022) investigated classifying the MRI acquisition sequence from single slice images of the brain using transfer learning on pre-trained CNN architectures, training three CNN models of varying complexity on a dataset of 113 brain MRI slices across four sequence types: T1-weighted with/without contrast, T2-weighted, and diffusion-weighted [12]. On an external testing set of 600 slices from 273 patients, their CNN models achieved categorical accuracy ranging from 79–84% in predicting the acquisition sequence [12]. Another relevant study by Ranjbar et al. (2019) developed a deep neural network with a variation of the Visual Geometry Group network (VGGNet) architecture that could classify four main brain MRI sequence categories—T1, T1 post-contrast, T2, and FLAIR—with 99% accuracy on a test set of 2,400 brain tumor patient images [13]. Liang et al. (2021) developed a random forest model that could identify the MRI sequence type for brain scans with 99.9% accuracy by using only the metadata encoded in the DICOM headers as input features, without analyzing the actual MRI image data itself [14]. Their approach demonstrated the ability to flag unknown sequence types based on the prediction confidence scores [14]. Vieira de Mello et al. (2021) used a CNN based on an 18-layer ResNet architecture to automatically classify volumetric brain MRI into sequence types like FLAIR, T1, T1 post-contrast, and T2, as well as identifying those that do not belong to any of those classes [15]. Evaluated on both pre-processed and non-pre-processed publicly available brain tumor datasets with diverse acquisition protocols, their CNN system achieved 96.81% accuracy in classifying the MRI sequence type [15]. Overall, these automated techniques leverage various image processing algorithms, machine learning models, and pattern recognition techniques to analyze MRI data comprehensively and categorize them according to their sequence types.

Contrary to earlier studies, this paper explores the development, implementation, and implications of a convolutional neural network architecture for identifying sequences, orientations, and contrast enhancement specifically in abdominal MRI. By automating the characterization of abdominal MRI sequences, this study can be of benefit for characterizing MRI studies across institutions and scanner types. This can be of particular benefit for large-scale, multi-institutional research projects, as this tool will organize MRI sequences collected using heterogeneous protocols. For instance, a multi-institutional study may specifically be looking to obtain T2-weighted, DWI, and ADC sequences for tumor analysis. These particular sequences can subsequently be analyzed either manually or using machine learning. MRI sequences are often labeled in an inconsistent manner, and manual curation of such datasets would be exceedingly time-consuming. This tool could also aid in the characterization of outside MRI scans, potentially as a sequence identification tool by PACS vendors. Finally, it could be used for the creation of MRI datasets organized by sequence either for research or quality control purposes.

## Methods

The data utilized in this research study was obtained from the University of Pennsylvania Health System, with the clinical information originating from patients who are participants in the Penn Medicine BioBank (PMBB). All participants in PMBB provided written informed consent for using their data upon biobank enrollment. The PMBB is IRB-approved by the University of Pennsylvania (IRB protocol 813,913). It is supported by the Perelman School of Medicine at the University of Pennsylvania, a gift from the Smilow family, and the National Center for Advancing Translational Sciences of the National Institutes of Health (CTSA award UL1TR001878). This research adhered to institutional guidelines and regulations.

The dataset encompasses a total of 26 distinct examinations from different patients. Each examination was identified by an 8-digit accession number, each containing multiple series of abdominal MRI scans with different sequences. In aggregate, 583 distinct abdominal MRI sequences were included in this study, encompassing a total of 29,484 images. However, it is important to note that 29,361 of these images were used in the analysis, as the remainder were discarded due to being corrupt DICOM files. The specific sequence type, orientation, and contrast of each MRI sequence were manually identified, and the findings were recorded in a comprehensive spreadsheet. Specifically, the MRI sequences were classified into 11 distinct categories, which include ADC, Bright Blood, Care Bolus, DWI, Dual Echo, Localizer, Subtraction, T1, T2, T2 Fat Saturation, and T2 MRCP Any MRI sequences that did not conform to one of these 11 categories were categorized as “Other.” Furthermore, the orientation of each MRI sequence was categorized as either axial, coronal, or sagittal. Sequences that did not align with any of these three orientation categories were classified as “N/A.” In addition to the sequence and orientation, the contrast utilization in the MRI sequences was assessed, indicating whether a particular sequence was acquired with or without contrast. Consequently, the total number of distinct classes within this dataset stands at twelve for sequences, four for orientation, and two for contrast.

Python 3.10 (Python Software Foundation, Wilmington, DE, USA) was the programming language of choice for processing the acquired abdominal MRI data and implementing machine learning techniques. The Google Colaboratory (Alphabet, Mountain View, CA, USA) integrated development environment was used. The primary libraries incorporated include Pydicom, Scikit-learn, Tensorflow, NumPy, and Matplotlib. The study workflow includes processing the data, building a convolutional neural network architecture specialized for the task, training and validating the model, and testing the model. The model takes single slices of abdominal MRI images as inputs, and it outputs predictions of their sequence type, orientation, and contrast enhancement.

### A. Pre-Processing

**Figure 1:**
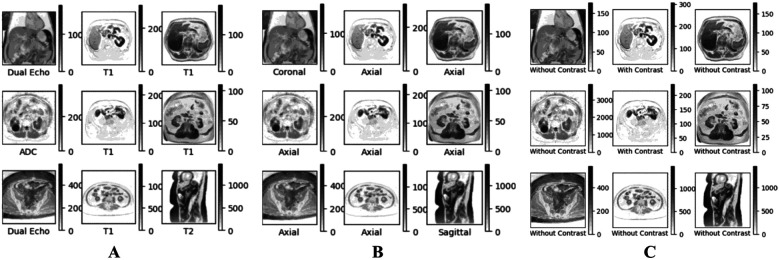
Sample sequence (A), orientation (B), and contrast (C) labeling of nine MRI image slices.

The abdominal MRI dataset originally consisted of 583 sequences and a total of 29,484 individual DICOM files. To ensure data integrity, each DICOM file underwent an initial examination to determine whether it contained usable pixel array data. Any corrupted image data was subsequently removed from the sequence. This process led to a dataset consisting of 583 sequences and 29,361 DICOM files. The unique series instance UID attribute for each MRI sequence was then employed to locate the corresponding sequence, orientation, and contrast labels within the curated spreadsheet. These labels were subsequently assigned to the MRI images within each sequence. Given the variations in image sizes and the number of channels, each MRI image underwent a standardization process. Specifically, all images were resized to 128 by 128 pixels and converted to grayscale. This uniform treatment resulted in a single 3-D pixel array with a shape of (29361, 128, 128), alongside three distinct 1-D arrays, each with a length of 29361, corresponding to the assigned sequence, orientation, and contrast labels respectively. Figure 1 shows the labeling of nine MRI image slices that were arbitrarily pulled from the dataset.

To prepare this data for training, the labels underwent one-hot encoding. A train-test split was subsequently carried out, allocating the first 80% of the data to the training set and reserving the rest 20% for the testing set. Given that the dataset was grouped by patients, this approach sought to prevent patient overlap between the training and testing sets, thereby ensuring that the model primarily undergoes evaluation on data from patients who were not encountered during the training phase. This partitioning resulted in a training dataset encompassing 23,488 images and a testing dataset of 5,873 images.

### B. Training

In this study, a convolutional neural network (CNN) was designed to effectively process and classify abdominal MRI images. The CNN architecture was meticulously constructed to capture intricate features within the data, enabling accurate multi-class classification of sequence and orientation, as well as binary classification of contrast. The neural network architecture consists of three distinct models, each tailored to address the classification tasks of sequence, orientation, and contrast.

**Figure 2:**
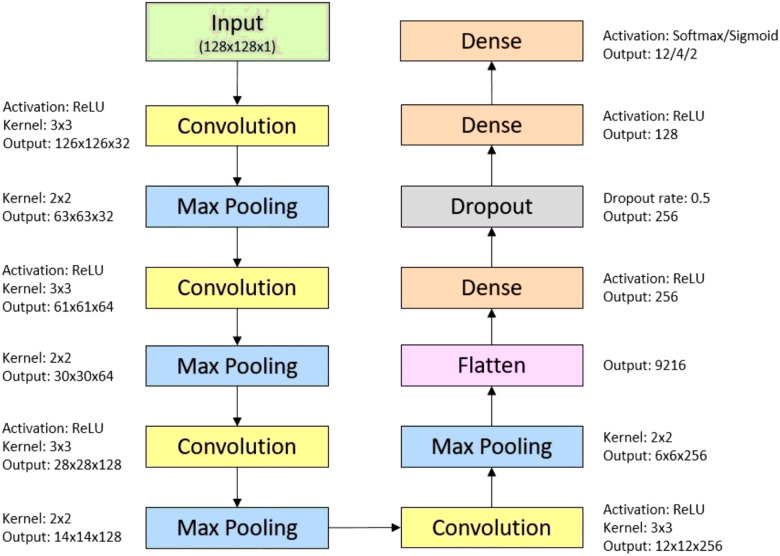
Convolutional neural network model architecture.

The first two models employ multiclass classification. They share a similar architecture, with the only notable difference being the number of output classes they are designed to classify. The first of these models is configured for a sequence classification task with twelve distinct output classes, while the second model is designed to handle an orientation classification with four distinct output classes. The model is structured as follows (Fig. 2). It begins with a 2D convolutional layer featuring 32 filters of size (3, 3) and a Rectified Linear Unit (ReLU) activation, tailored to accommodate grayscale images of dimensions (128, 128, 1). Following this initial layer, a max-pooling layer of size (2, 2) is introduced to reduce spatial dimensions. Subsequently, three successive convolutional layers with an increasing number of filters (64, 128, and 256) are employed to capture progressively abstract features, with the same max-pooling layer after each convolutional layer. Following these layers is a flatten layer that is applied to transform the feature maps into a one-dimensional vector, and three dense layers are incorporated into the model. The first dense layer, with 256 neurons and a ReLU activation, is followed by dropout regularization (50%) to prevent overfitting. The architecture includes two additional dense layers: one with 128 neurons, followed by a final dense layer that utilizes a softmax activation function. Depending on the multi-class classification task, there are 12 or 4 output neurons in this final layer.

The third model in our study is tailored for the binary classification of contrast. Unlike the multiclass models, this model employs a sigmoid activation in the final dense layer. The sigmoid activation is suitable for binary classification tasks, as it provides a probability score indicating the likelihood of a binary outcome.

The three models are trained using the Adam optimizer with a default learning rate of 0.001. The categorical cross-entropy loss function is employed to measure the disparity between predicted probabilities and actual class labels. The ‘accuracy’ metric is tracked during training to monitor the proportion of correctly classified samples. The models are each trained over 10 epochs, repeatedly exposing them to the entire training dataset. A batch size of 32 is used to process data efficiently, and 10% of the data is set aside for validation, enabling assessment of the model’s generalization to unseen data and preventing overfitting. Throughout this process, the model’s internal parameters are continually adjusted by the Adam optimizer to minimize the loss function. The model’s accuracy on both the training and validation data is recorded for each epoch.

### C. Testing

The final stage of this study involves comprehensively testing the developed models using the test set reserved during the pre-processing phase. Each model is independently evaluated by presenting it with these unseen images to analyze their proficiency in assessing sequence, orientation, and contrast. When each test image is processed through the models, it yields probabilistic outcomes ranging from 0 to 1 across various classes. These outcomes reflect the likelihood of an image belonging to a specific class.

Subsequently, these predictions are compared to the pre-established test labels through the computation of statistical metrics. Metrics including accuracy, precision, recall, and F1 score are calculated to quantify the model’s predictive capabilities. To offer greater insight into the performance, a visual aid in the form of a confusion matrix is constructed, displaying the agreement or discrepancy between the predicted classes and the true labels.

## Results

**Figure 3:**
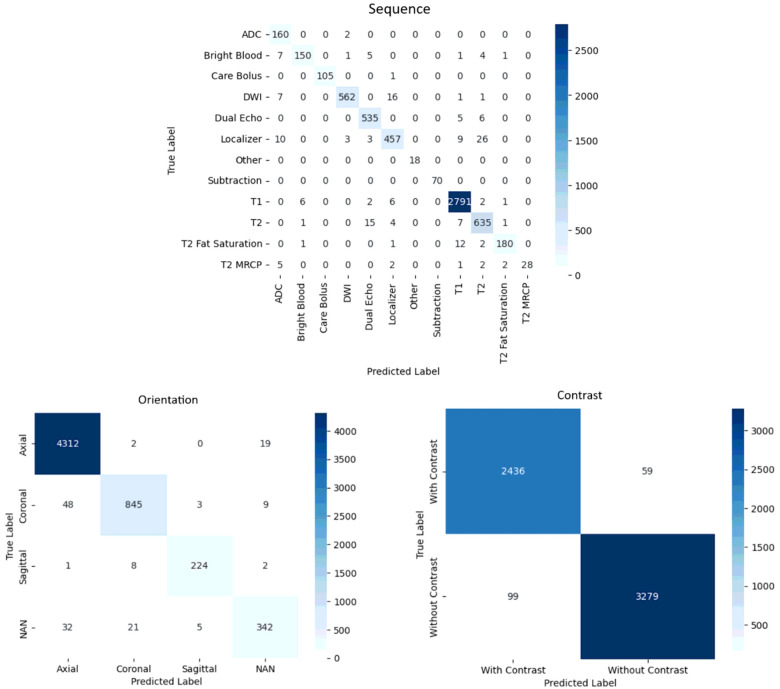
Confusion matrices for the classification of sequence, orientation, and contrast.

All three specialized CNN models designed for classifying sequence, orientation, and contrast demonstrated high performance. Across these classification tasks, the models demonstrated consistent and robust capabilities in accurately categorizing the MRI images. Specifically, the accuracies of each model for sequence, orientation, and contrast were 96.9%, 97.4%, and 97.3%, respectively. Detailed classification reports for the performance of each model are further displayed below, incorporating additional metrics such as precision, recall, and the F1 score (Table 1). Within a classification report, the support column represents the *true label count* within each class. The macro and weighted averages for precision, recall, and F1 score further provide a nuanced understanding of the results, factoring in support size for the weighted averages. In addition, the corresponding confusion matrices are shown (Fig. 3).

## Discussion

Overall, the convolutional neural network architecture employed in this study demonstrates robust performance for automatically characterizing abdominal MRI sequence, orientation, and contrast enhancement. Three similar, yet distinct, CNN models were developed, each optimized for sequence, orientation, and contrast classification tasks. By integrating appropriate combinations of convolutional layers, max-pooling layers, and dropout regularization, the models effectively captured intricate features while minimizing overfitting. Evaluation on a held-out test set confirms the high accuracies of the models for sequence, orientation, and contrast classification, demonstrating their robustness. These tailored algorithms showcase potential as a practical tool for automated and accurate MRI sequence characterization in abdominal imaging diagnostics.

Certain misclassifications, however, exist that are worth further examining and analyzing. The misclassification of MRI sequences underscores the complexity and ambiguity present in abdominal MRI data. Factors contributing to these misclassifications include the similarity in image features between different sequences and the intricate nature of the abdominal anatomy. Some notable misclassifications include T2 being predicted as Dual Echo, Localizer being predicted as T2, and DWI being predicted as Localizer. T2 and Dual Echo sequences often share similarities in signal intensity and tissue contrast, consequently leading to misclassifications by the model. Similarly, Localizer sequences, a brief set of images designed for patient positioning and scan planning, may exhibit anatomical landmarks resembling T2-weighted images, complicating differentiation. In addition, the lack of high image quality in localizer sequences compared to other sequences may point to why they were somewhat more likely to be confused, as evidenced by the confusion matrix.

Moreover, making predictions from a single image slice in MRI sequences poses limitations in capturing the entirety of the sequence characteristics and may lead to incomplete or inaccurate classification outcomes. With a sufficiently larger dataset size, training the neural networks to take in entire sequences instead of single images becomes feasible. Furthermore, adopting a majority voting approach could improve classification accuracy and robustness. By performing slice-by-slice classification within MRI sequences and aggregating predictions through majority voting, the model can derive a more comprehensive understanding of sequence content and assign the entire sequence to the most frequently predicted sequence type. This approach enhances the model’s ability to capture sequence dynamics and variability across multiple slices, thereby improving the fidelity and reliability of automated MRI interpretation in clinical practice.

In terms of future directions, it is essential to further consider the generalizability of the model’s performance to diverse patient populations, imaging protocols, and clinical settings. Robust validation across multiple healthcare institutions and datasets is essential to assess the model’s reliability and efficacy in real-world scenarios, ensuring consistency and accuracy in abdominal MRI interpretation across different clinical contexts. Although the dataset used in this study was from a single academic medical center with multiple hospitals that include urban and suburban environments, a future multi-institutional study could include more variability in patient demographics, imaging protocols, and pathologic conditions. To enhance the robustness and generalizability of the model, future research efforts should prioritize the acquisition of datasets from multiple healthcare institutions, encompassing diverse patient populations, imaging modalities, and clinical contexts. By incorporating datasets from various demographics and institutions, researchers can capture a broader spectrum of imaging characteristics and pathological presentations, fostering greater accuracy, generalizability, and reliability in MRI interpretation across different clinical settings.

## Conclusion

In this study, convolutional neural networks were used to classify single slices of abdominal MRI as one of 12 sequences, 4 orientations, and 2 contrast classes. Specifically, three CNN architectures were trained for separate sequence, orientation, and contrast classification tasks using similar backbone architecture. Model accuracies for sequence, orientation, and contrast were 96.9%, 97.4%, and 97.3%, respectively. The findings of this study underscore the potential of CNNs to automate and enhance the classification of abdominal MRI sequences. By leveraging deep learning techniques, it is possible to improve the efficiency, accuracy, and reliability of MRI characterization, ultimately facilitating large-scale research endeavors and fulfilling diverse clinical needs. While challenges and limitations persist, ongoing research efforts hold promise for addressing these obstacles and advancing the field of automated medical imaging interpretation.

## Figures and Tables

**Table 1.** Classification reports for sequence, orientation, and contrast.

Type	Category	Precision	Recall	F1 Score	Support
Sequence	ADC	0.85	0.99	0.91	162
	Bright Blood	0.95	0.89	0.92	169
	Care Bolus	1.00	0.99	1.00	106
	DWI	0.99	0.96	0.97	587
	Dual Echo	0.96	0.98	0.97	546
	Localizer	0.94	0.90	0.92	508
	Other	1.00	1.00	1.00	18
	Subtraction	1.00	1.00	1.00	70
	T1	0.99	0.99	0.99	2808
	T2	0.94	0.96	0.95	663
	T2 Fat Saturation	0.97	0.92	0.94	196
	T2 MRCP	1.00	0.70	0.82	40
	Macro-Avg	0.96	0.94	0.95	5873
	Weighted-Avg	0.97	0.97	0.97	5873
Orientation	Axial	0.98	1.00	0.99	4333
	Coronal	0.96	0.93	0.95	905
	Sagittal	0.97	0.95	0.96	235
	N/A	0.92	0.85	0.89	400
	Macro-Avg	0.96	0.93	0.95	5873
	Weighted-Avg	0.97	0.97	0.97	5873
Contrast	With Contrast	0.96	0.98	0.97	2495
	Without Contrast	0.98	0.97	0.98	3378
	Macro-Avg	0.97	0.97	0.97	5873
	Weighted-Avg	0.97	0.97	0.97	5873

## Data Availability

Due to the use of clinical data from a biobank in this study, public access to the dataset is not feasible. However, deidentified data may be made available to qualified researchers upon request, subject to the biobank’s regulations and the study’s IRB approval. For data access inquiries, please contact Hersh Sagreiya.
